# The *M. tuberculosis* Rv1523 Methyltransferase Promotes Drug Resistance Through Methylation-Mediated Cell Wall Remodeling and Modulates Macrophages Immune Responses

**DOI:** 10.3389/fcimb.2021.622487

**Published:** 2021-03-12

**Authors:** Sabeeha Ali, Aquib Ehtram, Naresh Arora, P. Manjunath, Deodutta Roy, Nasreen Z. Ehtesham, Seyed E. Hasnain

**Affiliations:** ^1^ Molecular Infection and Functional Biology Lab, Kusuma School of Biological Sciences, Indian Institute of Technology, New Delhi, India; ^2^ JH Institute of Molecular Medicine, Jamia Hamdard, New Delhi, India; ^3^ National Institute of Pathology, Safdarjung Hospital Campus, New Delhi, India; ^4^ Dr Reddy’s Institute of Life Sciences, University of Hyderabad Campus, Hyderabad, India; ^5^ Department of Biochemical Engineering and Biotechnology, Indian Institute of Technology, New Delhi, India

**Keywords:** MTases, antibiotics resistance, methyltransferases, cell envelope lipids, immune responses, *Mycobacterium smegmatis*, phage display

## Abstract

The acquisition of antibiotics resistance is a major clinical challenge limiting the effective prevention and treatment of the deadliest human infectious disease tuberculosis. The molecular mechanisms by which initially *Mycobacterium tuberculosis* (*M.tb*) develop drug resistance remain poorly understood. In this study, we report the novel role of *M.tb* Rv1523 MTase in the methylation of mycobacterial cell envelope lipids and possible mechanism of its contribution in the virulence and drug resistance. Initial interactome analyses predicted association of Rv1523 with proteins related to fatty acid biosynthetic pathways. This promoted us to investigate methylation activity of Rv1523 using cell wall fatty acids or lipids as a substrate. Rv1523 catalyzed the transfer of methyl group from SAM to the cell wall components of mycobacterium. To investigate further the *in vivo* methylating role of Rv1523, we generated a recombinant *Mycobacterium smegmatis* strain that expressed the Rv1523 gene. The *M. smegmatis* strain expressing Rv1523 exhibited altered cell wall lipid composition, leading to an increased survival under surface stress, acidic condition and resistance to antibiotics. Macrophages infected with recombinant *M. smegmatis* induced necrotic cell death and modulated the host immune responses. In summary, these findings reveal a hitherto unknown role of Rv1523 encoded MTase in cell wall remodeling and modulation of immune responses. Functional gain of mycolic acid Rv1523 methyltransferase induced virulence and resistance to antibiotics in *M. smegmatis*. Thus, mycolic acid methyltransferase may serve as an excellent target for the discovery and development of novel anti-TB agents.

## Introduction

The emergence of highly aggressive multi-drug-resistant (MDR) and extensively-drug-resistant (XDR) tuberculosis (TB) has been a major concern for the treatment of drug resistant TB (Chakaya et al., 2020). Therefore, there is a critical need to understand how antibiotics resistance is acquired and whether there is any connection between antibiotics resistance, virulence and aggressiveness. While multiple factors have been associated with drug resistance (Siddiqi et al., 2002), the methylome of the *M.tb* has recently been implicated to contribute to virulence and emergence of drug resistance (Shell et al., 2013; Warrier et al., 2016). Genes encoding MTases comprise of 3% of the *M.tb* genome (Grover et al., 2016). Methylation-specific modification(s) of different macromolecules of *M.tb* is considered to play an important and key role in survival of mycobacteria in the host, irrespective of local environmental pressures. The lineage specific methylation motifs in 4 prime lineages of *M.tb* and 2 lineage of *M. africanum* suggest a role of methylome in *M.tb* pathobiology (Phelan et al., 2018). Integrated genome-wide methylome and transcriptome studies identified several new MTases that cause antibiotic resistance. It is expected that the number of these modifying enzymes will increase with evolution (Dookie et al., 2018). We recently reported that *M.tb* methylome contains around 121 MTase genes (Grover et al., 2016). However, how the methylome of *M.tb* may participate in the pathogenesis and drug resistance is not fully understood.

The mycobacterial bacillus comprises of an exceptionally complex cell wall based on sugars and lipids of remarkable structure that set pathogenic mycobacterium aside from the non-pathogenic mycobacteria and most of other prokaryotes, and this also accounts for its pathogenesis (Alderwick et al., 2015). The essential components of cell envelope, mycolic acid, arabinogalactan, and peptidoglycan coordinate to form an mAGP complex of the mycobacterial bacilli which maintains a robust basal structure supporting the upper membrane. Arabinogalactan facilitates peptidoglycan to connect with the outer mycolic acid layer (Alderwick et al., 2015; Schwenk et al., 2018). Mycolic acids constitute a very high lipid component of the membrane and form the external mycomembrane to represent the cell envelope core composition that encloses the mycobacterial bacilli. The biosynthesis of mycolic acid in *M.tb* involves multifunctional enzyme complex FASI and FASII systems and also a variety of unique MTases (Takayama et al., 2005). Mycolic acid MTases (MAMTs) are crucial for *M.tb* survivability, virulence, integration of the cell wall complex, resistance to antibiotics (Rao et al., 2005; Takayama et al., 2005; Rao et al., 2006; Marrakchi et al., 2014; Alderwick et al., 2015). In the mouse model of infection, an *M.tb* deletion mutant of the proximal cyclopropane ring of α-mycolic acid or of methoxy- and ketomycolates results in restricted development of the two mutants. Similarly, *M.tb* deletion mutant of the keto-mycolates results in attenuation of development of the mutant inside the host cells (Rao et al., 2005; Rao et al., 2006). The emergence of MDR-, TDR-, and XDR-TB are suspected to be related to enzymes that are involved in the modification of the cell wall components, including enzymes implicated in synthesis and modification of mycolic acids (Ducati et al., 2006; Barry et al., 2007; Abrahams and Besra, 2018). Understanding the biosynthesis and assembly of these crucial decisive factors of the mycobacterial pathophysiology is critical for overcoming TB drug resistance. We describe here the characterization and function of a probable MTase coded by *M.tb* ORF*Rv1523*. This gene is only present in the pathogenic strains of mycobacterium (*M.tb* and *M. bovis*), suggesting its likely functional involvement in the pathophysiology of *M.tb* (Grover et al., 2016). Initial interactome analyses predicted association of Rv1523 with cell wall related proteins involved in fatty acid biosynthetic and mycolic acid biosynthetic pathways and it also has motif of mycolic acid cyclopropane synthetase domain. We investigated whether Rv1523 MTase can methylate the cell wall lipids, fatty acid or mycolic acid. Further, to understand the *in vivo* role in methylation, we expressed this ORF in *Mycobacterium smegmatis* strain, that potentially lacks *Rv1523* gene. Recombinant *M. smegmatis* strain expressing *Rv1523* exhibits altered cell wall lipid composition, remodeling the cell wall through methylation of mycolic fatty acid. Functional gain of mycolic acid Rv1523 methyltransferase increased *M.tb* virulence and resistance to antibiotics. Additionally, expression of *Rv1523* in *M. smegmatis* induced necrotic cell death of infected macrophages and modulated the host immune responses by decreasing the proinflammatory TNF-α and increasing the anti-inflammatory IL-10 production. These findings suggest Rv1523-mediated cell wall remodeling through methylation of mycolic acid may be responsible for acquiring resistance to antibiotics.

## Materials and Methods

### Bacterial Strains, Plasmids, and Culture

Middlebrook 7H9 medium, supplemented with 0.05% Tween80 and 0.2% glycerine was used to grow liquid culture *M. smegmatis* mc^2^155 strains and Middlebrook 7H10 plates supplemented with 10% glycerine were used to grow *M. smegmatis* colonies. *Escherichia coli* (*E. coli*) was grown in Luria-Bertani medium. Subsequent concentrations of antibiotics used were: hygromycin, 100 µg/ml; kanamycin, 50 µg/ml for *E. coli*, and 20 µg/ml for *M. smegmatis* mc2155. All *M. smegmatis* and *E. coli* cultures were incubated at 37°C with 160 rpm and 200 rpm, respectively. The bacterial strains, all the plasmids and the primer sequences used in this study are enlisted in **Table 1**.

### Cloning, Expression and Purification of Rv1523

The Rv1523 gene from *M.tb* was PCR amplified by using forward primer 5^′^-CGACATATGATGACGATAACCGCATTAAC-3^′^ and reverse primer 5^′-^GCCCTCGAGATCCTTGGCGAACAAG -3’. The PCR amplicons were digested with *Nde*I and *Xho*I and ligated into the *E. coli* expression vector pET28a. For mycobacterial expression, the full length Rv1523 was cloned in *Nde*I–*Hin*dIII sites of pVV16 mycobacterial shuttle vector. Rv1523 protein was purified according to standard procedure from *E. coli* BL21-DE3 strain (**Table S1**). For Rv1523 protein purification, secondary culture of recombinant *E. coli* BL21-DE3 expressing Rv1523 was set up in 250 ml LB broth with appropriate antibiotics. After reaching OD_600_ of 0.6–0.8, the culture was induced with 0.2mM IPTG and allowed to grow at 37°C, at 200 rpm for 4 h. The culture was harvested and the induced culture pellet was re-suspended in 25 ml of chilled lysis buffer of 50mM sodium phosphate containing lysozyme, PMSF, 0.1%–0.3% sarkosyl and kept on ice for 30 min. The suspension was sonicated for 10 min with regular on and off cycles of 10 s and 40 s each, respectively on ice and centrifuged at 12,000 rpm for 45 min Supernatant was collected separately and loaded on Ni-NTA agarose beads column. Washing was done with 40 ml of 30 mM and 40 mM imidazole solution in sodium phosphate buffer. Protein was eluted in gradient with 150–250 mM imidazole in sodium phosphate. Proteins of different sizes were separated on SDS-PAGE. For experimental purpose, the proteins were dialyzed in dialysis buffer (10mM sodium phosphate buffer, pH 7.8, 20 mM NaCl) and concentrated using 10 kDa cut-off membrane concentrator (Millipore, USA). Protein concentration was estimated by Bradford reagent.

### Construction of Recombinant *M. smegmatis* Expressing Rv1523

The plasmids (pVV16 and pVV16-Rv1523) were electroporated into *M. smegmatis* mc^2^155 (**Table S1**, **S2**) following protocols described by Wards and Collins (2016). The recombinant *M. smegmatis* strains were selected on 7H10 medium containing 20 ug/ml kanamycin and 100 ug/ml hygromycin. The recombinant Ms_Rv1523 and Ms_Vec were subjected to colony PCR and western blotting.

### Detection of the Expression of Rv1523 in *M. smegmatis*

Liquid cultures of both the recombinant *M. smegmatis* strains Ms_Vec and Ms_Rv1523 were grown in 7H9 broth medium until the OD_600_ reached 0.6 to 0.8. The bacterial cell fractionation of recombinants Ms_Vec and Ms_Rv1523 was performed as mentioned previously, with minor modifications. Conventionally both the recombinant mycobacterial cultures were harvested using centrifugation at 6,000 rpm for 10 min at 4°C. After washing the cell-lysate with PBS, it was sonicated in cold PBS supplemented with protease inhibitor. After sonication, cell lysate was centrifuged at 11,000 rpm into the insoluble pellets and the soluble supernatant fractions. Both the fractions were loaded to SDS-PAGE and subsequently identified by Western blot experiment using specific anti-1523 polyclonal antibody. The blots were developed by incubating with IgG-HRP, anti-mouse IgG antibody labelled with horseradish peroxidase.

### Production of Anti-Rv1523 Antibodies

A 500 µg/ml of purified Rv1523 protein was used to immunize a rabbit. At an interval of 15 days, 3 booster doses of immunization were given. Finally, 15 days after the last immunization the sera were collected.

### *In-Silico* Analysis of Rv1523

Using the bioinformatic STRING (Search Tool for the Retrieval of Interacting Genes _version 9.1) (http://string.embl.de), the interacting associates for Rv1523 protein from the *M.tb* H_37_Rv genome database were identified. All the parameters used for the STRING interaction assessment were set as default. The prediction methods selected were all active: i.e., database, gene fusion, neighbourhood, co-expression, co-occurrence, experiments, and text mining. The S-score (confidence score) set to a default mid-range 0.4 was used to predict the probability of association between the two proteins. A medium confidence level corresponds to 50% possibility of an interaction. Each node indicates a protein, and each edge represents an association in the displayed network. Protein motif and domain analysis of Rv1523 was performed using InterProScan analysis (http://www.ebi-.ac.uk/interpro/).

### Phage Display Assay

The assay was performed as mentioned in the protocol with some minor modifications (Ph.D.™-7 Phage Display Peptide Library Kit (New England Biolabs Inc.). Surface preparation: Target solution 150µl in 0.1M NaHCO_3_, pH.6 was applied to each well and incubated overnight at 4°C. Coating solution was poured off and blocking buffer added and incubated for at least 1 h at 4°C. Blocking solution was discarded and washed with TBST six times. One round of selection: The Phage library (10 µl) was diluted into 100 µl in TBST. Phages were pipetted onto target-coated surface and phages and target were incubated together with gentle mixing for 2–3 h. After binding, non-binding phages were discarded and washed with TBST ten times. To elute the bound phages 100ul of elution buffer (0.2M glycine-HCl, pH 2.2, 1 mg/ml BSA) were added and phages were eluted. The elution buffer was further neutralized with 20µl of 1M Tris-HCl, (pH 9.1).

Phage Enrichment: An overnight culture of ER2738 was diluted 1:100 in 10 ml LB in 60 ml glass tube. All but 20 µl of eluted phage pool was added. The culture was set at 200 rpm for shaking at 37’C for 6 h. The culture was centrifuged at low rpm and 9 ml of phage containing supernatant was transferred to a clean centrifuge tube. Phages were precipitated by adding 3 ml of 2.5M NaCl/20%PEG-8000 and incubated on ice overnight. The sample was centrifuged at 11,000 rpm for 20 min. Supernatant was removed and the off-white phage pellet was gently resuspended in 1 ml TBS. After 10 min incubation the residual cells were centrifuged at 11,000 rpm. The phages were re-precipitated by adding ⅙ volume of NaCl/PEG and incubated on ice for 1 h and then centrifuged for 20 min at 11,000 rpm. The supernatant was removed, and the phage pellet was resuspended in 200µl TBS.

Phage Titration: The ER2738 cells were inoculated in 10 ml of LB and kept on shaking at 37°C for 4–8 h until mid-log phase, till OD_600_ of ~0.6 was reached. Phage stock was diluted in 1 ml volume of LB with 10-10^3^-fold serial dilutions to yield ~10–500 plaques per plate. 10ul of phage dilutions were added to 200 ul of ER2738 culture of OD_600_ of ~0.6, and allowed 1 min for infection to take place, infection time was kept constant with each of the phage dilution. The entire infection volume was transferred into warm top agar tubes, vortexed briefly and solution was poured onto pre-warmed LB/IPTG/XGal plate and incubated overnight at 37°C. The pfu/ul was calculated and additional rounds of selection were carried out using 10^11^ pfu for input for each round. Three rounds were carried out before proceeding with sequencing and binding studies.

### Extraction and Purification of Mycobacterial Cell Wall Fatty Acid Methyl Esters (FAMEs) and Mycolic Acid Methyl Esters (MAMEs)

Mycobacterial cell wall fatty acid and mycolic acids was purified following protocols as described (Dupont and Kremer, 2014). The liquid mycobacterial cultures of *M. smegmatis* were inoculated and grown, in Middlebrook 7H9 broth and incubated at 37°C with slow shaking till the OD_600_ reached (OD_600_ = 1) of the culture. Exponentially growing mycobacteria were harvested for mycolic acid extraction. The mycobacterial pellet was resuspended in 4 ml of tetrabutylammonium hydroxide (TBAH) and incubated overnight at 100°C for hydrolysis of complete fatty/mycolic acid. To the 4 ml of TBAH mixture, 8 ml CH_2_Cl_2_, 600μl CH_3_I and 4 ml water were added and mixed well for 1.5 h at room temperature for methyl esterification of the mycolic acids. It was centrifuged at 3,500 rpm for 10 min at room temperature to separate the mixture in two phases: a lower organic phase containing the lipids and an upper aqueous phase that was discarded. The tube was mixed for 25 min after adding 5 ml of water and centrifuged at 3,500 rpm for 10 min at room temperature. The wash step was repeated three more times and the upper phase was removed. The lower organic phase was dried, 4 ml diethyl ether was added, and sonication was performed in a water bath for 5–10 min at room temperature. It was further centrifuged at 3,500 rpm for 10 min at room temperature and methyl esters were transferred in a new glass tube. The diethyl ether was evaporated under a stream of nitrogen and the residue was resuspended in 100–200 µl CH_2_Cl_2_. The extracted fatty acid methyl esters (FAMEs) and mycolic acid methyl esters (MAMEs) were resolved by thin layer chromatography using hexane/ethyl acetate (19:1, v/v). The TLC plate was sprayed with 5% ethanolic molybdophosphoric acid, lipid bands corresponding to the different mycolic acids revealed following charring using a heat gun.

### Methyltransferase Assay

The methylation assay was performed using 50 ng protein, mycolic acid (6µl) and 6.9mM of S-adenosyl methionine (SAM) for 5 min (or as otherwise indicated) in assay buffer (10 mM Tris pH 7.4, 1 mM dithiothreitol (DTT). Both the enzyme and the substrate in combination was titrated in the assay to determine optimal conditions. The SAM MTase assay buffer + additive was equilibrated to 37°C (SAM510™: SAM Methyltransferase Assay - G-Biosciences, Cat. # 786‐430). A total volume of 5μl of purified SAM MTase enzyme was taken in duplicate in a 96 well plate. The SAM MTase assay buffer or 0.1M Tris, pH 8.0 was used as a diluent. The reactions and controls were performed in duplicates. For the background control, 5 μl SAM MTase assay buffer was aliquoted into each background control well. The assay is supplied with Adenosylhomocysteine enzyme as a positive control.

5 μl positive control and 10 μl SAM MTase assay buffer was added to each positive control well. The extracted mycolic acid (acceptor substrate) was added (6 ul) to the sample and background control wells, using SAM MTase assay buffer. The reaction was initiated by adding 100 μl SAM MTase master mix to the wells. The wells were zeroed, and absorbance was measured at 510 nm collecting data every 1–2 min at 37°C until the increasing absorbances plateau (60 min).

### Extraction and Resolution of Mycobacterial Cell Wall Mycolic Acids

The recombinant mycobacterial strains Ms_Vec and Ms_Rv1523 were inoculated in 7H9 medium till OD reached OD_600_ >1. The bacterial cells were harvested by centrifugation and treated with tributyl-ammonium hydroxide (TBAH) for alkaline hydrolysis of bacterial surface, as described previously. The extracted mycolates were finally resuspended in dichloromethane prior to TLC analysis. Briefly, equal amounts of each sample were applied to a silica-coated aluminium TLC plate, which was developed in different mixtures of solvents depending on the nature of the TLC.

### Treatment with Anti-Tubercle Drug TAC Analogue, SRI-224

The recombinant mycobacterial strain Ms_Rv1523 was grown in 7H9 medium till OD_600_ reached 0.8. TAC analogue SRI-224 solutions were added to the growth medium at desired concentrations of 0 ug, 5 ug, 10 ug, for a total period of 48 h. Cells were harvested, followed by methyl-esterification, extraction, and resolution of mycolic acids by thin layer chromatography (TLC) as described previously.

### HPLC Conditions

An Agilent 1200 infinity series HPLC (Agilent Technologies, 1260 infinity, Open Lab, CS) equipped with a Zorbex Eclipsed C-18 reverse-phase analytical cartridge column (SB-C18, 4.6 mm by 15.0 cm packed with 5 μm silica) was used for chromatographic separation of the cell wall components using a gradient of 98% phase A (95% methanol and 5% methylene chloride) and 2% phase B (50% acetonitrile and 50% methanol) with a flow rate of 0.8 mL min-1. During the first minute following the injection, the solvent concentrations were changed to 80% phase A-20% phase B, and then changed linearly at the 5 min to 50% phase A-50% phase B. During the next 35 min the column was re-equilibrated in 80% phase A-20% phase B. The total run time was 50 min.

### *In Vitro* Survival Under Different Anti-Microbial Stresses

The growth patterns of both the recombinant *M. smegmatis* cultures were analyzed in 7H9 broth media consisting 100 μg/ml hygromycin and grown up to OD_600_ = 0.8. In the presence of surface stress and pH stress the growth curves were plotted against culture time for Ms_Vec and Ms_1523. For surface stress, culture of Ms_Vec and Ms_1523 was exposed to 0.05% SDS for a duration of 1, 2, 3, and 4 h. For acidic pH stress, pH gradient was produced by supplementing concentrated HCl in 7H9 broth medium and the pH of media was adjusted to pH 3 and pH 5. The media was further filter sterilized by passing through a 2 μm filter. After SDS and acid stress exposure, both the recombinant strains Ms_Vec and Ms_Rv1523 were diluted and plated into 7H10 agar consisting hygromycin for bacteria quantification.

### Anti-Tuberculosis Drug Sensitivity Assays

Nine antibiotics were used in this study, including vancomycin (Van), oflaxacin (Ofl), norfloxacin (Nor), rifampicin (Rif). The recombinant strains Ms_Vec and Ms_Rv1523 were grown till OD_600_ reached 0.06 to 0.08, the bacterial culture was diluted 2-fold and 50µl of each strain was plated into 7H10 agar medium and the HiComb™ MIC Test strip was placed on the culture containing different concentration of the antibiotic. HiComb™ MIC Test strip provides a set of 16 different concentrations in gradient that can be easily used to deduce a functionally accurate the Minimum Inhibitory Concentration (MIC) in microgram levels. MIC values of each antibiotic were assessed by analysing the bacterial growth after 3 days culture.

### Macrophage Infection Assay

Macrophages infection assay was performed using RAW264.7 cells (1 × 10^5^/well) seeded in 24-well plates in complete DMEM media and incubated overnight at 37C, 5% CO_2_. Before infection, recombinant Ms_Vec and Ms_1523 were passaged 4–5 times through a 26-gauge needle to disperse aggregated cells and then macrophage cells were infected at an MOI of 10:1. Infection was allowed for a total time period of 2 h at 37°C, 5% CO_2_, after which macrophage cells were washed thoroughly with PBS and gentamicin (20 μg/ml) was added to kill the extracellular bacteria and incubated for a further 0, 4, 24, and 48 h at 37°C, 5% CO_2_. The infected macrophage cells were washed at each time point with PBS and the macrophages lysed with 1 ml 0.01% SDS to release the intracellular mycobacteria. The mycobacterial cells were plated onto 7H9 agar supplemented with the appropriate antibiotic to enumerate the CFU.

### Cytokines Production Analysis

PMA-differentiated THP-1 cells cultured in RPMI media (Invitrogen) were infected with Ms_Vec and Ms_Rv1523 at an MOI of 10. After 6, 24, and 48 h infection, culture supernatants were harvested, and the cytokines released by infected cells in the culture supernatants were quantitatively detected using ELISA kits of Tumor Necrosis Factor-α (TNF-α Invitrogen™ 88-7346-88) and interleukin-10 (IL-10 Invitrogen™ 88-7106-88).

### Lactate Dehydrogenase (LDH) Activity Assay and Detection of NO Production

Murine macrophages RAW 264.7 infected with Ms_Vec and Ms_Rv1523 for 2 h at 37°C, 5% CO_2_ and macrophages cells without infection were used as control. For lactate dehydrogenase (LDH) activity analysis, the culture supernatants from infected cells and control cells without infection were harvested after 24 and 48 h after infection. Following the standard protocols, the LDH activities were assessed using commercially available LDH cytotoxicity kit (Thermo). By using a Griess reagent (Thermo) which measures the stable end product, nitrite, the NO levels in culture supernatant of infected macrophages were determined. An equal volume of 100 µl of Griess reagent and culture supernatant was added to a 96-well plate in duplicate and incubated at room temperature for 15 min. Using standard curve for nitrite, levels of nitrite was estimated after taking absorbance at 540 nm. The results were showed as the mean µmoles of nitrite/sample ± SEM.

### Statistical Analysis

Results were expressed as the mean ± SEM of at least three independent experiments.

GraphPad Prism 9 software was used for statistical analysis. ANOVA (for two groups) and Kruskal-Wallis tests (for more than two groups) were used to determine statistical significance. A *p* < 0.05 was considered significant, *p < 0.05, **p < 0.01, ***p < 0.001, and ****p < 0.0001 denote the level of significance.

## Results

### Probable Interacting Partners of Rv1523 MTase

To identify the function of Rv1523 MTase, *M.tb.* proteins that could possibly interact with the Rv1523 was identified using phage display assay and STRING (Search Tool for the Retrieval of Interacting Genes/Proteins), followed by motif analysis by InterProScan. Phage display assay which detects ligands or interacting partners (Wu et al., 2016) uses bacteriophages expressing a library of random heptapeptides (7 amino-acid containing peptide), on their cell surface as fusion to the coat protein (pIII) of M13 phage. The displayed peptide is expressed at the N-terminus of pIII. The purified Rv1523 protein (**Figure S2**) was immobilized and used as a target, and heptapeptides ligand sequences showing interaction with Rv1523 protein were extracted and identified by DNA sequencing (**Figure S1**, **Table S3**). After 3 rounds, the proteins containing these ligand heptapeptides were explored by *in silico* BLAST approach using the ligand peptide as a query sequence. The sequences of the protein showing >70% interaction, i.e., five out of seven amino acids, with the ligand sequences, were taken for further analysis. The ligand sequences ADARYKS, HWNTVVS, DRGHHIL, MPRLPPA, and AWPYVTL displayed interaction with Rv1523 in the second round and ISTTLFP, NTALSST, ADARYKS, LQKGMT, SHLNVHS, ASSHIHH, NGATYPS, and ADARYKS showed interaction in third round. Rv1523 interacting protein partners included the following: NADPH-dependent 2,4-dienoyl-CoA reductase, fatty acid biosynthesis transcriptional regulator, putative ftsk/spoiiie family protein, lysophospholipid transporter lplt, ESX-4 secretion system protein eccd4, carbon monoxide dehydrogenase, O-antigen/lipopolysaccharide transport integral membrane protein rfbd, sugar ABC transporter permease, carbohydrate ABC transporter membrane protein CUT1 family, Acyl-CoA dehydrogenase fadE1, fatty-acid-CoA ligase, outer membrane protein icsa autotransporter precursor, glycine dehydrogenase [decarboxylating], aconitate hydratase, Acyl-CoA dehydrogenase fadE24. The majority of these Rv1523 interacting proteins (>70% of the total interactome) belong to two families, 1) cell cycle protein and, 2) fatty acid biosynthesis/metabolism (**Table 1**). A careful inspection of the target protein pathways of these two families converge on a common pathway of fatty acid biosynthetic and mycolic acid biosynthesis pathways.

**Table 1:** List of possible interacting ligands, the functional category of each and a summary of the functional pathway or reaction catalyzed.

S.N.	Interacting partners	Functional category	Functional pathway
1.	**NADPH-dependent 2,4-dienoyl-coa reductase**	Fatty acid biosynthesis/metabolism	metabolism of unsaturated fatty enoyl-CoA esters
2.	**Fatty acid biosynthesis transcriptional regulator**	Fatty acid biosynthesis/metabolism	transcriptional regulation of Fatty acid biosynthesis/metabolism
3.	**Putative ftsk/spoiiie family protein**	Cell wall and cell processes	A transmembrane protein, involved in cell division processes
4.	**Lysophospholipid transporter lplt**	Cell wall and cell processes	Catalyzes the facilitated diffusion of 2-acyl-glycero-3-phosphoethanolamine (2-acyl-GPE) into the cell
5.	**ESX-4 secretion system protein eccd4**	Cell wall and cell processes	involved in transport across the membrane
6.	**Carbon monoxyde dehydrogenase**	Fatty acid biosynthesis/metabolism	It catalyses the interconversion of CO and CO2 and the synthesis of acetyl-coenzyme
7.	**O-antigen/lipopolysaccharide transport integral membrane protein rfbd sugar ABC transporter permease**	Fatty acid biosynthesis/metabolism	Involved in the biosynthesis of the dTDP-L-rhamnose which is an important component of lipopolysaccharide
8.	**Carbohydrate ABC transporter membrane protein CUT1 family**	Cell wall and cell processes	Contributes to cuticular wax and suberin biosynthesis. Involved in both decarbonylation and acyl-reduction wax synthesis pathways. Required for elongation of C24 fatty acids, an essential step of the cuticular wax production https://www.uniprot.org/uniprot/Q9XF43
9.	**Acyl-CoA Dehydrogenase fadE1**	Fatty acid biosynthesis/metabolism	Catalyzes the dehydrogenation of acyl-coenzymes A (acyl-CoAs) to 2-enoyl-CoAs, the first step of the beta-oxidation cycle of fatty acid degradation.
10.	**Fatty-Acid-CoA ligase**	Fatty acid biosynthesis/metabolism	that activates the breakdown of complex fatty acids
11.	**Outer membrane protein icsa autotransporter precursor**	Cell wall and cell processes	Essential for bacterial spreading by eliciting polar deposition of filamentous actin
12.	**Isocitrate dehydrogenase**	Fatty acid biosynthesis/metabolism	Catalyzes the oxidative decarboxylation of isocitrate, producing alpha-ketoglutarate (α-ketoglutarate) and CO2
13.	**Glycine dehydrogenase [decarboxylating]**	Fatty acid biosynthesis/metabolism	Glycine cleavage system and Lipoic acid formation which is an essential cofactor for the oxidative decarboxylations of α-keto acids
14.	**Aconitate hydratase**	Fatty acid biosynthesis/metabolism	Aconitate hydratase is the mitochondrial form of aconitase, an enzyme that catalyses the stereo-specific isomerization of citrate to isocitrate *via* cis-aconitate in the tricarboxylic acid cycle.
15.	**Acyl-CoA dehydrogenase fadE24**	Fatty acid biosynthesis/metabolism	Acyl-CoA dehydrogenases (ACADs) are a class of enzymes that function to catalyze the initial step in each cycle of fatty acid β-oxidation

**Figure 1:**
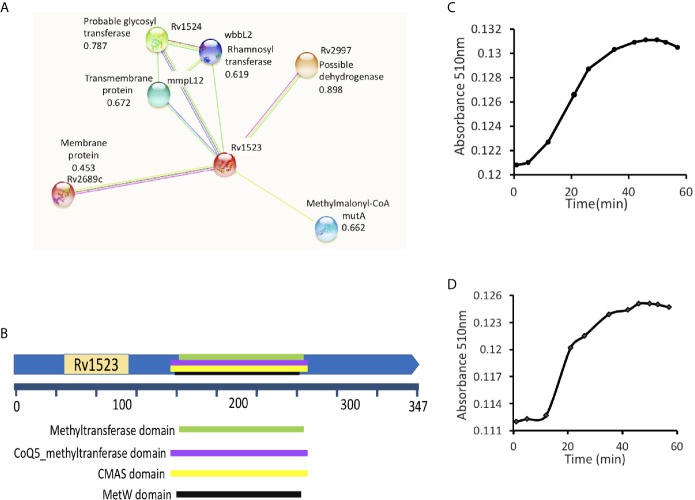
Rv1523 is a fatty acid MTase. **(A)** STRING analysis reveals the top 10 interaction partners, both known and putative of *M. tuberculosis* H*37*Rv Rv1523 protein. The score for each interaction partner is assigned and given. The score for Rv1523 association was found to be 0.672 for mmpL12, a transmembrane protein, 0.787 for a probable glycosyltransferase and 0.898 for dehydrogenase enzyme. **(B)** Functional domain analysis of *M. tuberculosis* Rv1523 protein. The InterProScan analysis result shows the presence of Mycolic acid cyclopropane synthetase domain (CMAS) other than MTases domain, SAM-binding domain, MetW domain, CoQ5_methyltranferase domain. **(C)** The Adenosylhomocysteine methyltransferase enzyme (provided with the kit) was used as a positive control. Methyltransferase enzymatic activity analysis of Rv1523 was analyzed along with positive control, as a function of time, in a colorimetric based assay. **(D)** Rv1523 methyltransferase activity was measured in terms of increase in absorbance. Purified cell wall components - fatty acid methyl esters (FAMEs) and mycolic acid methyl esters (MAMEs) were used as a substrate for estimation of Rv1523 enzymatic activity, the absorbances was plotted against time in a colorimetric based assay.

Using STRING, an *in silico* tool, possible binding partners of Rv1523 protein were identified. The Rv1523 protein showed interaction with several enzymes/proteins as their interacting partner (**Figure 1A**). The following four proteins - methylmalonyl-CoA (possible dehydrogenase), hypothetical protein (possible methyltransferase), rhamnosyl transferase (probable glycosyl transferase) and one transmembrane protein showed interaction with Rv1523 protein. The STRING database also predicted one of functional partners of Rv1523 as a conserved transmembrane protein - MmpL12. The Mycobacterial membrane protein large (MmpL) proteins export substrates that are required for mycobacterial membrane synthesis and directly support the ability of the pathogen to infect and persist in the host. Deletion or chemical ablation of MmpL3 activity reduces the transport of mycolic acid across the plasma membrane and further decreases cell membrane mycolylation (Melly and Purdy, 2019). These interacting partners provided a clue on the possible role of Rv1523 in cell wall synthesis related functional pathway.

Further FASTA sequences of Rv1523 was subjected to domain analysis using an *in silico* approach InterProScan. This software predicted the presence of mycolic acid cyclopropane synthetase domain (CMAS) (160–260 amino acids) along with MTase domain (165–259 amino acids), SAM-binding domain (157–263 amino acids), MetW domain (165–259 amino acids), CoQ5_methyltranferase domain (160–260 amino acids) (**Figure 1B**). Together these findings point to a likely connection between Rv1523 and biosynthesis/modification pathways of cell wall fatty acid and mycolic acid.

### Rv1523 Functions as a Mycolic Fatty Acid MTase

Interactome analyses showing the presence of mycolic acid cyclopropane synthetase domain motif in Rv1523 promoted us to investigate methylation activity of Rv1523 using cell wall component fatty acids- FAMEs and MAMEs as substrate. The enzymatic activity of the Rv1523 MTase was investigated using cell wall component fatty acids to mimic *in vivo* conditions. To determine the methyl transfer enzymatic activity of Rv1523, *in vitro* system was constituted comprising of purified recombinant Rv1523 protein, SAM as the methyl group donor and cell wall component fatty acid esters, and the rate of Rv1523 MTase activity was measured as a function of time, in a colorimetric based assay with a colorimetric reagent, 3,5‐dichloro‐2‐hydroxybenzenesulfonic acid (DHBS). The enzymatic activity of Rv1523 was analyzed along with positive control, adenosyl-homocysteine MTase enzyme which was part of the MTase enzyme activity analysis kit (**Figure 1C**). An increase in absorbance at 510 nm indicated that Rv1523 MTase was able to transfer methyl group from SAM to the cell wall component fatty acids of mycobacterium (**Figure 1D**). Rv1523 methyltransferase activity was also expressed as activity µmol/min/ml. The change in Rv1523 enzyme activity with time was studied using 50 ng Rv1523 protein, cell wall component fatty acid esters as substrates and SAM as methyl group donor in a colorimetric based assay (**Figure S3A**). Taken together, these results showed novel fatty acid methylating activity of Rv1523 MTase.

### Expression of Rv1523 in the Non-Pathogenic Mycobacteria *M. smegmatis* Remodels the Cell Wall Lipid Components

The distinctive cell wall lipid composition is a characteristic feature of mycobacteria, which influences diverse mycobacterial phenotypes. The subtypes of mycolic acid and their relative ratios differ with the mycobacterial strain. *M. smegmatis* is devoid of methoxy and keto-mycolic acid and synthesizes two types of mycolates: alpha- and oxygenated epoxy- mycolates. Therefore, *in vivo* functional role of Rv1523 in mycobacterium cell wall lipid composition was determined by generating Rv1523 overexpressing *Mycobacterium smegmatis* by cloning Rv1523 gene in mycobacterial shuttle vector pVV16 using *Nde*I and *Hin*dIII restriction sites, and successful transformation of pVV16_1523 was confirmed by colony PCR (**Figure S3B**). The recombinant strain Ms_Rv1523 showed the expression of Rv1523 gene whereas no expression was detected in vector alone (Ms_Vec) by western blot analysis (**Figure S3C**).

**Figure 2:**
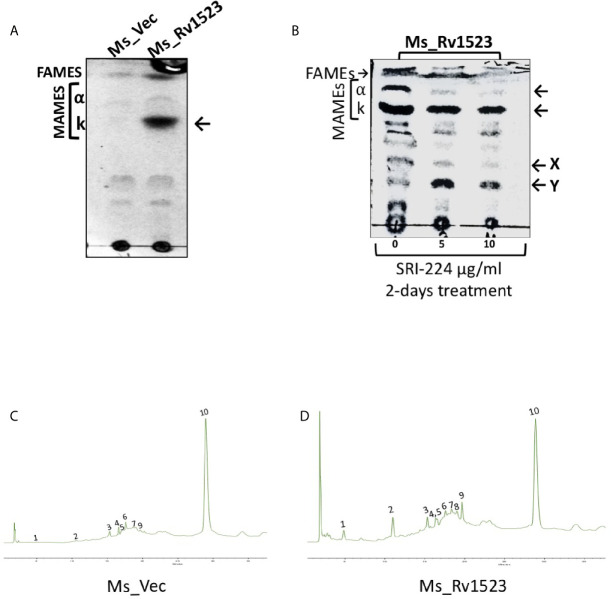
Rv1523 MTase is a cell wall remodeler. **(A)** Exponentially growing cultures of Ms_Vec and Ms_Rv1523 were taken and fatty acid methyl esters (FAMEs) and mycolic acid methyl esters (MAMEs) were then extracted and separated by TLC. All extracts were loaded equally. The chromatogram shows FAMEs, MAMES, α- and keto-mycolates (k). TLC analysis was carried out using hexane and diethyl ether (19:1, v/v) as solvents. **(B)** Mycolic acid biosynthesis in *M. smegmatis* expressing Rv1523 is inhibited after treatment with TAC analogue SRI-224. 1D TLC profile of FAMEs and MAMEs, extracted from cells of recombinant Ms_Rv1523, treated with SRI-224 for 2 days, with different concentrations, as indicated. The chromatogram shows FAMEs, MAMEs, α- and keto-mycolates (k) as indicated by arrowheads and the possible lipids X and Y mentioned by (Alahari et al, 2007). **(C)** Exponentially growing cultures of Ms_Vec and Ms_Rv1523 were taken and fatty acid methyl esters (FAMEs) and mycolic acid methyl esters (MAMEs) were then extracted and analyzed by HPLC. 20 µl of each sample were injected for analysis. HPLC chromatograms of the cell wall component of recombinant Ms_Vec. **(D)** Ms_Rv1523 showing significant difference in peak 1, 2, and 9 confirming the change in cell wall lipid profile of Ms_Rv1523 as compared to Ms_Vec.

Further, it was examined whether the expression of Rv1523 in recombinant *M. smegmatis* affected the cell wall lipid composition. The methyl-esters of the cell envelope as FAMEs (fatty acid methyl esters) and MAMEs (mycolic acid methyl esters) were prepared followed by TLC profiling. The mycolic acid profiles of recombinant *M. smegmatis* with and without expression of Rv1523 gene revealed that there was an increased amount of a possible keto mycolates in *M. smegmatis* expressing Rv1523, but not in the pVV16 vector alone transformants (**Figure 2A**).

To further confirm the role of Rv1523 in mycolic acid biosynthesis, we examined the effects of an inhibitor of mycolic acid synthesis - SRI-224 on the production of alpha- and keto mycolic acids. SRI-224 is an analogue of anti-tubercle drug thiacetazone which specifically inhibits the cell wall components - α-mycolates as well as keto-mycolates (Alahari et al., 2007). Recombinant Ms_Rv1523 cells, upon treatment with different concentration of SRI-224, showed a major decrease in the amount of α-mycolic acid and keto-mycolic acid. In contrast, as expected there was an increase in the precursors of the α- and keto-mycolates in recombinant Ms_Rv1523 cells treated with the inhibitor-SRI-224. In summary, these findings indicate that the *M. smegmatis* strain expressing Rv1523 exhibited altered cell wall lipid composition, particularly keto mycolates (**Figure 2B**).

### HPLC Analysis of Rv1523 Expressing Recombinant *M. smegmatis* Cell Wall Components

Classically, the separation of cell wall extracts with chromatographic techniques followed by reverse-phase HPLC analysis is used to demonstrate the chemical nature of heteropolysaccharides from *M.tb* strains and to distinguish the pathogenic slow-grower and non-pathogenic rapid-grower mycobacterium strains (Hagen and Thompson, 1995; Butler and Guthertz, 2001). The methyl-esters of the cell envelope as FAMEs (fatty acid methyl esters) and MAMEs (mycolic acid methyl esters) of the recombinant Ms_Rv1523 and vector control Ms_Vec were subjected to reverse-phase UV-HPLC analysis with an altered gradient elution system of chloroform-methanol to differentiate chromatographically comparable categories of the Ms_Rv1523 and Ms_Vec. Multiple factors affected the separation of mycolic acid in the reverse-phase HPLC column, including the functional group, carbon chain length, and polarity. The derivatization of mycolic acid to UV-absorbing esters increased the sensitivity of hydrocarbon detection (Butler and Guthertz, 2001). Comparison of the fatty acid profiles of the Ms_Rv1523 with that of the Ms_Vec showed that Ms_Vec was devoid of peak 1, 2, and 9 (**Figures 2C, D**), whereas several fatty acid peaks appeared at significantly higher levels in Ms_Rv1523 transformant. These fatty acids peaks were either not detected or present in low levels in the Ms_Vec controls. Based on the above findings, it is tempting to speculate that the difference in the HPLC profiling between Ms_Rv1523 and vector control Ms_Vec cell wall components may be a consequence of Rv1523 gene overexpression on mycolic acid synthesis and altered cell envelope components.

### Expression of Rv1523 Confers Resistance to Recombinant *M. smegmatis* Against Several Anti-Tuberculosis Drugs

**Figure 3:**
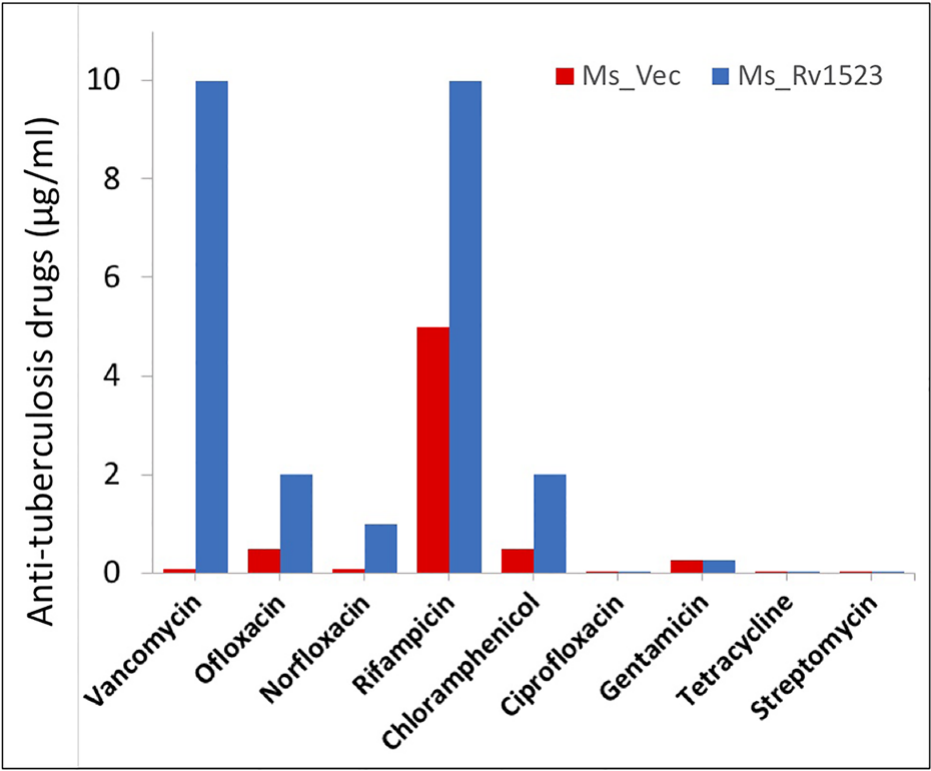
Ms_Rv1523 was resistant to anti-tuberculosis drugs. Recombinant Ms_Rv1523 and Ms_Vec were treated with nine anti-tuberculosis drugs and MIC of nine antibiotic was detected. The MIC values of Ofl, Rif, Van, and Nor for Ms_Vec were 0.5 μg/ml, 5 μg/ml, 0.1 μg/ml, and 0.01 μg/ml while 2 μg/ml, 10 μg/ml, 10 μg/ml, and 0.1 μg/ml for Ms_Rv1523, respectively. The MIC values of Ms_Rv1523 for Van and Nor were 100 and 10 fold higher than Ms_Vec, respectively.

The cell wall of *M.tb* is meant to provide an efficient barricade and prevent entry of different antibiotics (Brennan and Nikaido, 1995; Smith et al., 2013). Therefore, we evaluated whether the modifications in cell envelope of Ms_Rv1523 at all impact cell envelope permeability. Recombinant Ms_Rv1523 and Ms_Vec were exposed to nine anti-TB drugs as mentioned in the methods and the MIC was identified (**Table 2**). Both Ms_Rv1523 and Ms_Vec showed equivalent susceptibility to ciprofloxacin (Cip), gentamicin (Gen), tetracycline (Tet), and streptomycin (Str). The MIC values of Cip, Gen, Tet and Str for Ms_Vec and Ms_Rv1523 were 0.001 μg/ml, 0.256 μg/ml, 0.1 μg/ml, and 0.001 μg/ml, respectively (**Figure S4**). However, Ms_Rv1523 showed remarkable resistance to ofloxacin (Ofl), rifampicin (Rif), especially vancomycin (Van) and norfloxacin (Nor). The MIC values of Ofl, Rif, Van, and Nor for Ms_Vec were 0.5 μg/ml, 5 μg/ml, 0.1 μg/ml, and 0.01 μg/ml as opposed to 2 μg/ml, 10 μg/ml, 10 μg/ml, and 0.1 μg/ml for Ms_Rv1523, respectively. The MIC values of Ms_Rv1523 for Van and Nor were 100 and 10 fold higher than Ms_Vec, respectively (**Figure 3**). These observations point to a novel role of MTase family protein Rv1523 in resistance to anti-TB drugs.

**Table 2:** Drug sensitivity to anti-tuberculosis drugs.

Anti-tuberculosis drugs (μg/ml)	Ms_Vec	Ms_Rv1523
**Vancomycin (Van)**	0.1	10
**Ofloxacin (Ofl)**	0.5	2
**Norfloxacin (Nor)**	0.1	1
**Rifampicin (Rif)**	5	10
**Chloramphenicol (Chl)**	0.5	2
**Ciprofloxacin (Cip)**	0.001	0.001
**Gentamicin (Gen)**	0.256	0.256
**Tetracycline (Tet)**	0.01	0.01
**Streptomycin (Str)**	0.001	0.001

### Rv1523 Expressing Recombinant *M. smegmatis* Was More Resistant to Antimicrobial Stress Factors

**Figure 4:**
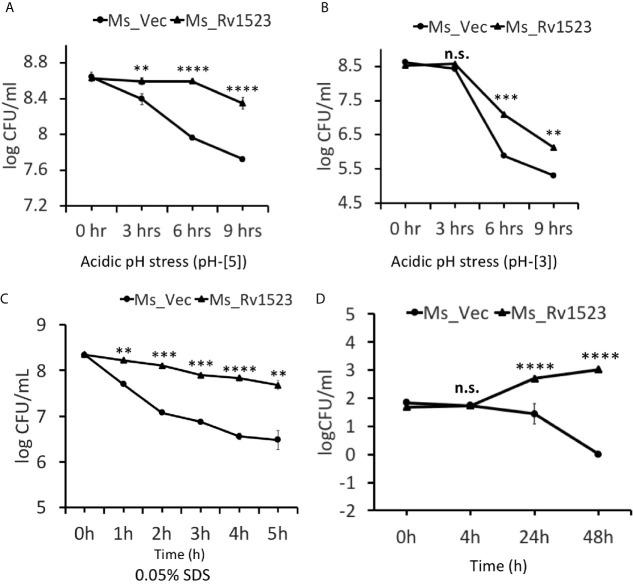
Rv1523 expression in *M. smegmatis* causes resistance to antimicrobial stress factors. The growth of Ms_Vec and Ms_Rv1523 after treatment, for various times, with different pH gradient pH-[3] **(A)** and pH-[5] **(B)**. Cultures of Ms_Vec and Ms_Rv1523 were harvested, re-suspended to 5 ml 7H9 media at an OD_600_ of 0.6, 10-fold serial dilutions of Ms_Vec and Ms_Rv1523 were spotted on 7H10 agar plates. Ms_Rv1523 was more resistant to acidic pH stress at both the pH gradients pH-[3] and pH-[5] as compared to Ms_Vec. **(C)** Ms_Rv1523 was observed to be ~100–1,000 fold resistant to the surface stress as compared to Ms_Vec. For surface stress analysis mid-log-phase cultures of Ms_Vec and Ms_Rv1523 were incubated in 7H9 media supplemented with 0.05% SDS for indicated time. The recombinant strains were plated onto 7H10 agar plates after serial ten-fold dilution. Bacterial numbers were counted after 3–4 days of growth. **(D)** Expression of *M. tuberculosis* Rv1523 in *M. smegmatis* increased 100–1,000 fold intracellular survival within macrophage. Mouse RAW264.7 cells were infected with Ms_Vec and Ms_Rv1523 at an MOI of 10:1. After 4, 24, and 48 h infection, macrophages were washed and lysed using 0.01% SDS. Lysates were plated on 7H10 medium to enumerate the CFU count. Two-way ANOVA was used for calculating statistical significance. All the experiments were performed at least three times; SEM is represented by error bar for biological triplicates. ns, non-significant, **p < 0.01, ***p < 0.001, and ****p < 0.0001.

The role of mycolic acid MTases (MAMTs) is widely recognized in *M.tb* survivability, integration of the cell wall complex, and resistance to antibiotics (Rao et al., 2005; Takayama et al., 2005; Rao et al., 2006; Marrakchi et al., 2014; Alderwick et al., 2015). Having shown that recombinant Ms_Rv1523 was more resistant to many anti-TB drugs, such as vancomycin, rifampicin, ofloxacin and norfloxacin, we analyzed the role of Rv1523 in growth under conditions of acid and surface distress. As shown in **Figures 4A, B**, the survival of Ms_Rv1523 was significantly higher as compared to control Ms_Vec after 6 and 9 h exposure to acid stress (pH 3 and pH 5). The acid sensitive mutants of *M.tb* were also reported to be hypersensitive to several other stresses such surface stress, oxidative stress, and antibiotics stress (Vandal et al., 2009; Dookie et al., 2018). Accordingly, to rule out whether Rv1523 has any role in surface stress, Ms_Vec and Ms_Rv1523 were treated with 0.05% SDS that simulated surface distress. The survival of Ms_Rv1523 was 10- to 100- fold higher compared to that of Ms_Vec when treated with SDS for specified time, indicating highly resilient cell membrane in Ms_Rv1523 (**Figure 4C**). These results suggest that Rv1523 promotes resistance of *M. smegmatis* to surface and acid stress by possibly strengthening cell wall integrity and permeability.

### Expression of Rv1523 Enhanced Cell Survival of *M. smegmatis* Within Macrophages

Several cell wall modifying enzymes are required for pathophysiology of *M.tb*, which also promote the ability of the bacilli to grow inside the macrophages (Forrellad et al., 2013; Bussi and Gutierrez, 2019). To understand the role, if any, of Rv1523 in *M.tb* virulence we performed macrophage infection experiments and compared the intracellular survival of Ms_Vec and Ms_Rv1523. Recombinant Ms_Vec and Ms_Rv1523 were used to infect murine macrophages cell line (RAW264.7 cells) at an MOI of 10:1 at 37°C for 4 h, followed by washing with PBS and gentamycin treatment to kill extracellular bacteria. The number of intracellular bacilli was counted by enumerating the CFU at distinct time periods post infection. A significant difference in the survival of bacteria was observed in murine macrophages between Ms_Rv1523 and Ms_Vec at 24 h after infection (**Figure 4D**). To confirm that the increased survival is not a result of variation in infection rate of the two strains, the entry of mycobacteria inside the macrophages was measured immediately after 4hr infection at 37°C. The bacterial CFU recovered from macrophages were enumerated after washing the extracellular bacteria several times. No difference in the infection rate of Ms_Vec and Ms_Rv1523 was observed. These results indicated that recombinant *M. smegmatis* expressing Rv1523 showed enhanced survival in macrophages, pointing to a role of Rv1523 in bacterial virulence and persistence.

### Rv1523 Promotes Macrophage Cell Death

**Figure 5:**
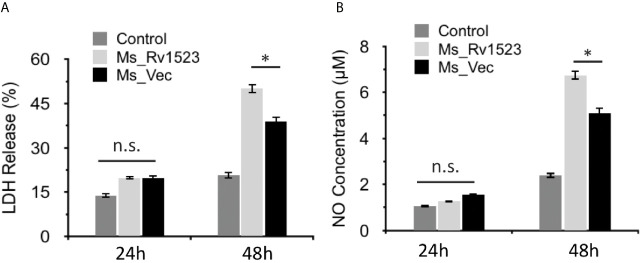
Expression of *M. tuberculosis* Rv1523 in *M. smegmatis* promotes death of macrophages. **(A)** The release of LDH was observed to be more in Ms_Rv1523 infected macrophages; and similarly levels of NO released **(B)** were higher in the culture supernatants of Ms_Rv1523 infected macrophages as compared to the culture supernatants of Ms_Vec infected macrophages suggesting that Rv1523 expression induces and promotes necrotic cell death of the macrophages. Macrophages were infected with Ms_Rv1523 (white bars) or Ms_Vec (black bars) at an MOI of 10:1. No infection was used as control (gray bars). After 24 and 48 h infection, culture supernatants were collected and the levels of LDH released and the levels of NO released were measured. Data are shown as means ± SEM of triplicate wells. Kruskal–Wallis test was used for calculating statistical significance. ns, non-significant *p < 0.05.

Induction of apoptosis, a programmed cell death, generally favors the host cells by affecting the intracellular pathogen viability and by increasing host immunity (Molloy et al., 1994; Duan et al., 2016). However, *M.tb* has been reported to manipulate apoptosis cell death pathway for dissemination and infection progression (Dallenga et al., 2017). To gain insight into whether Rv1523 has any role in bacterial dissemination *via* necrosis of infected cells, the release of LDH (Chan et al., 2013) into the culture supernatants was determined after infection with both, Ms_Vec control and Ms_Rv1523. The levels of LDH released were significantly higher in the case of Ms_Rv1523 infected macrophages as compared to Ms_Vec infected macrophages following 24 h infection (**Figure 5A**). These results indicate that Rv1523 expression in *M. smegmatis* induces necrotic cell death of macrophage as evident from release of LDH.

### Infection with Ms_Rv1523 Increased NO Production

The progression of infectious diseases is associated with increased levels of nitric oxide (NO) production which further triggers either necrotic or apoptotic cell death (Abebe et al., 2011; Tundup et al., 2014). We accordingly investigated the level of NO released into the culture supernatants of Ms_Vec and Ms_Rv1523 infected macrophages. The levels of NO released were higher in culture supernatants of Ms_Rv1523 infected macrophages compared to Ms_Vec after 24 h infection (**Figure 5B**). These results further indicated that Rv1523 induced NO production by infected macrophages.

### Rv1523 Modulates Macrophage Immune Responses

**Figure 6:**
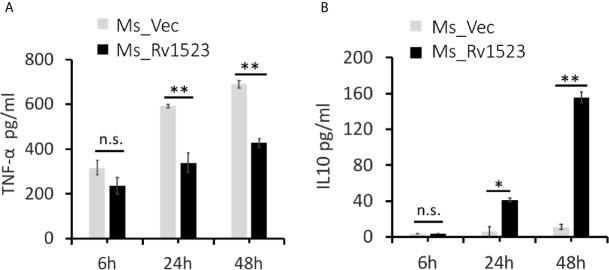
Rv1523 modulates macrophage immune responses. Overexpression of *M. tuberculosis* Rv1523 in *M. smegmatis* modulates the immune pathway by suppressing production of pro-inflammatory cytokine and inducing anti-inflammatory cytokine production. PMA-differentiated THP-1 cells (2 × 106/well/ml) were infected with recombinant Ms_Vec and Ms_Rv1523 strains. After 6, 24, and 48 h infection, the infected cells and supernatant were collected. ELISA was used for detecting the production of TNF-α **(A)** and IL-10 **(B)**. Experiments were performed at least twice; SEM is represented by error bar for biological triplicates. Two-way ANOVA was used for determining statistical significance. ns, non-significant *p < 0.05, and **p < 0.01.

Pro-inflammatory cytokines are considered necessary to control bacterial infection (Romero-Adrian et al., 2015; Blanc et al., 2017). One such pro-inflammatory cytokines TNF-α, is crucial for mounting an early pro-host immune response upon *M.tb* infection (Khubaib et al., 2016; Blanc et al., 2017; Sharma et al., 2018). To analyse the functional involvement of Rv1523 in the early immune responses, PMA-differentiated ThP-1 macrophages were infected with Ms_Vec or Ms_Rv1523 for 6, 24, and 48 h. After infection, the supernatants were collected at different time point for assessment of cytokine production using ELISA. The levels of pro-inflammatory cytokine TNF-α secretion was observed to be remarkably lower in macrophages infected with Ms_Rv1523 than Ms_Vec (**Figure 6A**). However, macrophages infected with Ms_Rv1523 produced higher levels of anti-inflammatory cytokine IL-10 compared to Ms_Vec infected macrophages (**Figure 6B**). IL-10 suppresses the anti-mycobacterial immunity by inhibiting the expression of host-protective pro-inflammatory cytokines and promotes the survival of pathogen. A decrease in levels of TNF-α, accompanied by increased IL-10 cytokine levels was consistently observed in Ms_Rv1523 infected macrophages. These results suggest that Rv1523 could be possibly playing a role in regulating cytokine production and immune modulation favoring the pathogen.

## Discussion

The Rv1523 gene is a part of *Mycobacterium tuberculosis* H_37_Rv’s genome that shows significant similarity with other MTases and was hence categorized as a probable MTase. However, the exact function of Rv1523 remains to be understood. Despite not having an established substrate for its catalytic function to date, this gene has been associated with pathogenicity of mycobacterium. Rv1523 gene is present and is transcribed in *M.tb* lineages, but not in non-pathogenic bacteria, therefore, it has been thought to be involved in *M.tb* virulence. Also, the appearance of Rv1523 protein in *in vivo* proteomics of the tubercle bacillus in the guinea pig model of tuberculosis after 90 days post infection and its absence during initial phases of infection (30 days post infection) point to its role in important biological processes for establishment of a productive bacterial infection and its persistence (Kruh et al., 2010). But, how Rv1523 methyltransferase enzyme may contribute in the virulence of mycobacterium remains to be understood.

In the current study, the role of Rv1523 methyltransferase enzyme in *M.tb* virulence and physiology was analyzed using *in silico* and *in vitro* approaches. *In vitro* phage display assay and *in silico* InterproScan and STRING analysis predicted the possible role of Rv1523 in *M.tb* cell wall component biosynthesis or modification. The unique cell wall composition of mycobacteria is important for the pathophysiology of *M.tb* and is also involved in resistance against anti-mycobacterial factors, and anti-tuberculosis drugs (Abrahams and Besra, 2018; Dookie et al., 2018). The cell wall of pathogenic mycobacteria is composed of distinctive heteropolysaccharides which include covalently linked mycolic acids, arabinogalactan and peptidoglycan and contribute to maintaining the integrity of the mycobacterial cell envelope and essential for the viability of *M.tb* (Alderwick et al., 2015). The cell wall lipids of pathogenic mycobacteria are extremely inflammatory and could stimulate the host immune reactions (Sheikh et al., 2020). The cell wall lipids mycolyl-arabinogalactan-peptidoglycan (mAGP) complex is critical for mycobacterial virulence and pathophysiology and a variety of unique glycosyltransferases and MTases are used for its biosynthesis and assembly (Takayama et al., 2005). The production of cell wall heteropolysaccharide is the result of multifunctional protein FAS-I and multienzyme complex FAS-II. Unlike the FAS-I protein which is a single large multidomain protein, the FAS-II pathway is composed of multiple individual proteins including lipid MTases working in complex to carry out reactions such as condensation, keto-reduction and dehydration of long chain fatty acid (Takayama et al., 2005; Marrakchi et al., 2014). While the FAS-I protein was found exclusively in the 30-day samples, the members of the FAS-II pathway were found primarily in the 90-day samples (Kruh et al., 2010). Our findings of functional partner analysis as well as reconstitution of *in vitro* MTase suggest that Rv1523 is a putative mycolic acid MTase (MAMT) that may be involved in cell wall fatty acid biosynthesis/metabolism in the FAS-II pathway.

We were tempted to analyse the functional pathway and identify and demonstrate the role of MTase catalytic domain by performing site directed mutation analyses of Rv1523 enzyme. However, our study of Rv1523 motif/domain prediction, using *in silico* tool InterProScan indicated the localization of the overlapping catalytic domains of Rv1523 enzyme spanning from 155 to 260 amino acids (**Figure 1B**). Deletion of a whole domain of approximately 100 amino acids residues for functional characterization, would therefore disturb the folding and structural configuration of the enzyme which will itself impact its function. We are therefore realized that studies involving truncated Rv1523 would not confirm whether the difference in the pathogenesis of the mycobacteria is due to the deletion of catalytic domain or due to the structural variation of the enzyme. Hence, the *in vivo* functional role of Rv1523 was investigated by using a widely used non-pathogenic mycobacterium surrogate bacillus, *M. smegmatis*. No significant difference in the growth rate of recombinant strains, Ms_Rv1523, and Ms_Vec was observed. We also did not observe any morphological changes between these two recombinant strains of *M. smegmatis* namely, Ms_Rv1523, and Ms_Vec, under bright field microscope using Ziehl-Neelsen (AFB Staining) technique. However, it was found that Rv1523 could significantly alter the cell wall lipid composition as compared to Ms_Vec transformant, suggesting a potential role of Rv1523 in cell wall fatty acid biosynthesis/metabolism. The changes in cell wall of recombinant Ms_Rv1523 might affect the function of mycobacterial cell wall such as, increased resistance to SDS and acid stress implicating a role of Rv1523 in stress response. Further, treatment with various anti-tuberculosis antibiotics propounded that Ms_Rv1523 is more resistant to antibiotics including anti-tuberculosis drug rifampicin, vancomycin, norfloxacin, and a second line anti-tuberculosis drug ofloxacin. Rifampicin, a broad spectrum antibiotics which is used against bacterial pathogens, is one of the key component of first-line drug treatment of anti-TB therapy and a marker for multi drug resistance (Siddiqi et al., 2002). Rifampicin diffuses effectively in mycobacterial cells and shortened the duration of TB treatment. The mode of action of rifampicin involves inhibition of bacterial DNA-dependent RNA polymerase blocking the transcription, and elongation of RNA (Campbell et al., 2001). Interestingly, mode of action of vancomycin is through inhibition of cell wall synthesis by binding to the D-Ala-D-Ala terminal of the growing peptide chain (Hammes and Neuhaus, 1974; Sarkar et al., 2017). This results in inhibition of the transpeptidase enzyme, thereby preventing elongation and cross-linking of the peptidoglycan matrix. This inhibition weakens bacterial cell walls and ultimately causes leakage of intracellular components, resulting in bacterial cell death (Patel et al., 2020). The mechanism of action of Norfloxacin, ofloxacin and other quinolone/fluoroquinolone antimicrobials depends on blocking of bacterial DNA replication, transcription, repair and recombination by binding itself to bacterial topoisomerase IV and DNA gyrase (both of which are type II topoisomerases) (Aldred et al., 2014). Ofloxacin and other piperazine-containing quinolones, such as norfloxacin has also been reported recently as an active compound against cell wall related transporters like autolysin. It was observed that ofloxacin binds to choline-binding domain of cell membrane transporters through its piperazine domain (Fernández-Tornero et al., 2005). Taken together these results lend support to the argument that the functional site of action of Rv1523 is the mycobacterial cell wall. Rv1523 modifies the properties of mycobacteria cell envelope when expressed in *M. smegmatis*, and these modifications are reflected in terms of the ability to change the cell wall lipid composition which in turn affects the cell wall integrity and permeability. In future studies it will be interesting to speculate on the possible role of Rv1523 in biofilm formation. The Rv1523 mediated changes in the cell wall composition and permeability of Ms_Rv1523 may also explain the intracellular survival of Ms_Rv1523.

The fatty acid composition and arrangement of mycobacterial cell wall is an important defence against local environmental pressures (Abrahams and Besra, 2018). Many acid-sensitive mutants showed defective cell wall function, and also sensitive to antibiotics and other stresses. The site of action of MAMT or mycolic acid cyclopropane synthetase (CMAS) domain containing enzymes is the proximal or distal chain of the long chain of mycolic acid that has to pack after folding at the site of the motifs (mycolic unit, cyclopropane, and keto group) to fit in a conventional membrane of 7–8 nm in thickness (Zuber et al., 2008). Formation of cyclopropane ring by MAMT or keto-reduction fits the long chain of the mycolic acid into the small space of cell wall and make it tightly and densely packed.

Our findings also highlighted immune response induced by Rv1523 that may help in survival of *M.tb.* Macrophages are the first line of host immune defence against *M.tb* infection (Woo et al., 2018) but *M.tb* employs multiple strategies to counteract the host immunity, thereby successfully establishing a niche within macrophages (Mahamed et al., 2017). Recombinant *M. smegmatis* expressing Rv1523 showed higher survival within the RAW264.7 murine macrophages than Ms_Vec. The enhanced intracellular survival within the macrophage can also be credited to the enhanced resistance to the stresses evaluated, as recombinant *M. smegmatis* expressing Rv1523 is more resistant to *in vitro* anti-mycobacterial stresses.

The essential components of the host immune defence against mycobacterial infection are the immunoregulatory molecules, such as TNF-α, IL-6, and IL-1β (Romero-Adrian et al., 2015; Blanc et al., 2017). The interaction with *M.tb* cell wall effectors and host can modulate the expression of pro-inflammatory and anti-inflammatory cytokines to benefit its survival inside the host macrophages (Esin et al., 2013; Stanley and Cox, 2013). Available data also indicate that lipid MTases by changing the cell wall composition play an important role in subverting innate immune responses and thus help the pathogen in establishment of infection (Rao et al., 2005). Recombinant Ms_Rv1523 modulated immune response in THP-1 macrophage cell lines by suppressing the production of pro-inflammatory cytokines and increasing the anti-inflammatory cytokine production. Strategically, once *M.tb* establishes itself in host macrophages by subverting innate immune defences it can then modulate the adaptive host immune mechanism for its survival (Karakousis et al., 2004; Hanna Wakim and El Beyrouthy, 2014). Apart from immune modulation, many lipid MTases enzymes have also been implicated in phagosome maturation block (pcaA) which is an important host defence mechanism for killing of these bacteria (Corrales et al., 2012).

**Figure 7:**
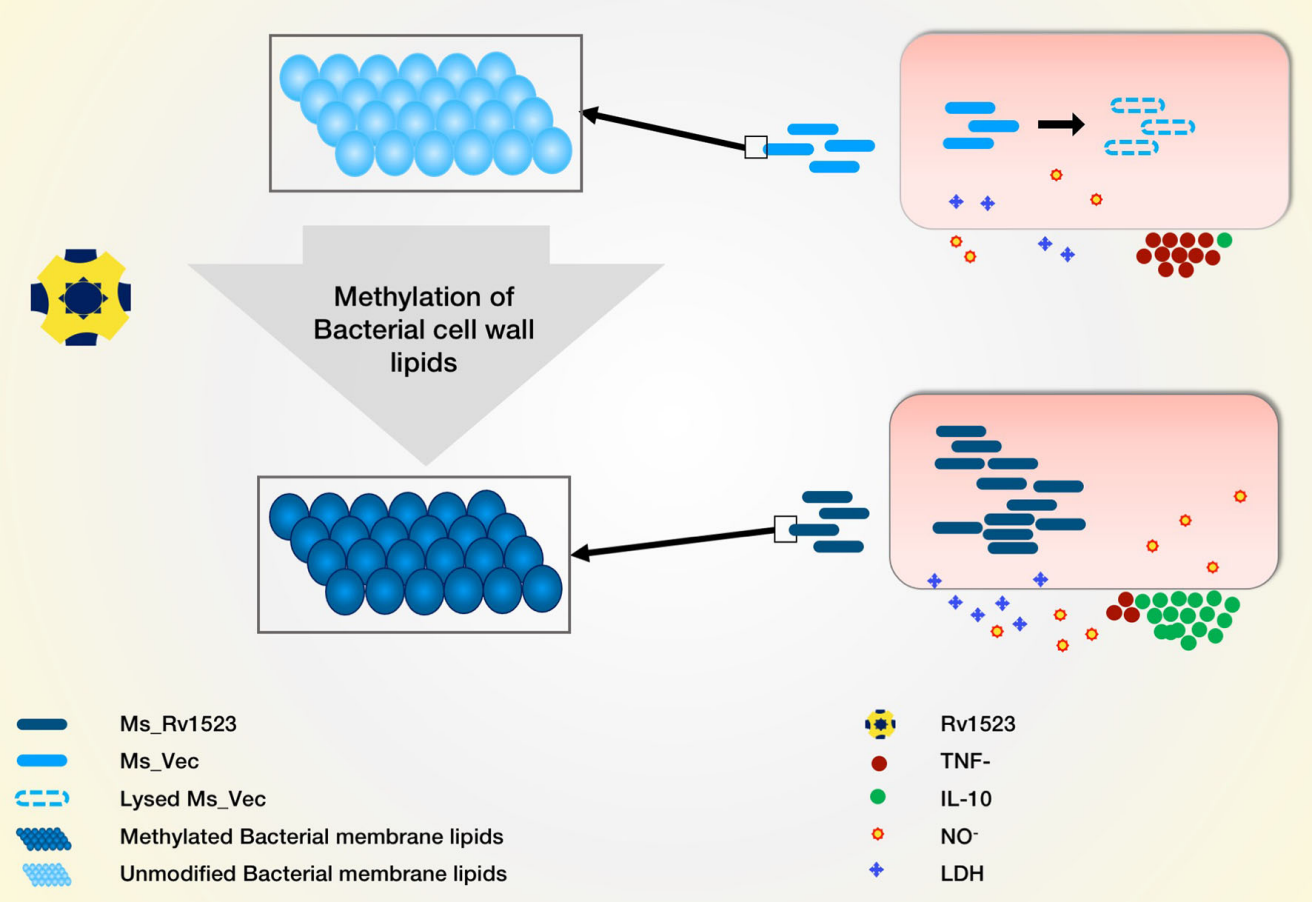
Model depicting the role of Rv1523 in bacterial cell wall lipid modulation and macrophages immune response alteration. We predict the role of Rv1523 methyltransferase in cell wall lipid modulation. Recombinant *M. smegmatis* expressing Rv1523 (Ms_Rv1523) displayed higher survival in macrophages as compared to *M. smegmatis* not expressing Rv1523 (Ms_Vec). Ms_Rv1523 induced increased expression of anti-inflammatory cytokine IL-10 and reduced the expression of proinflammatory TNF- α.

In summary, our results suggest that Rv1523 MTase is important for maintaining a resilient cell wall structure and has immune-modulatory effect which enables the bacteria to persist longer inside the macrophages (**Figure 7**). The expression of Rv1523 in the nonpathogenic *M. smegmatis* adds specific properties, including altered cell wall integrity, and cell wall remodeling. This is the first report of Rv1523 being directly or indirectly involved in mycolic fatty acid metabolism. Additionally, a role for Rv1523 in the stability and integrity of mycobacterial cell walls was evident from the increased sensitivity of the recombinant *M. smegmatis* to acid stress. The possible localization of *M. tuberculosis* Rv1523 at the cell envelope and the involvement of this protein in mycobacterial cell wall alteration may likely be responsible for active modulation of the host immune response. The functional role of cell wall MTases in pathophysiology of *M.tb* makes this Rv1523 mycolic acid MTases enzyme an attractive target for development of novel drugs against TB.

## Data Availability Statement

The original contributions presented in the study are included in the article/**Supplementary Material**. Further inquiries can be directed to the corresponding authors.

## Author Contributions

The study was designed by SH, NE, and SA. SA, NA, AE, and PM carried out the experiments. SA was helped by SH and DR in writing the manuscript. All authors contributed to the article and approved the submitted version.

## Funding

This work was supported by a Centre of Excellence grant (BT/PR12817/COE/34/23/2015) from the Department of Biotechnology (DBT), Ministry of Science and Technology, Government of India. SH is a JC Bose National Fellow, Department of Science and Technology (DST), Government of India, and Robert Koch Fellow, Robert Koch Institute, Berlin, Germany. SA is a recipient of a Senior Research Fellowship from Department of Biotechnology, Government of India. AE is a recipient of a Senior Research Fellowship from CSIR India. DR is a 2019-2020 Fulbright-Nehru Academic and Professional Excellence Scholar at Jamia Hamdard Institute of Molecular Medicine, New Delhi. The lab facilities at Jamia Hamdard were supported by DST PURSE Grant.

## Conflict of Interest

The authors declare that the research was conducted in the absence of any commercial or financial relationships that could be construed as a potential conflict of interest.
