# Supplementary material for: The M. tuberculosis Rv1523 Methyltransferase Promotes Drug Resistance Through Methylation-Mediated Cell Wall Remodeling and Modulates Macrophages Immune Responses
Source: Front Cell Infect Microbiol. 2021 Mar 12;11:622487. doi: 10.3389/fcimb.2021.622487 (PMC7994892; doi:10.3389/fcimb.2021.622487)
Supplement: Supplementary file 1 [file DataSheet_1.pdf]

Supplementary Figures

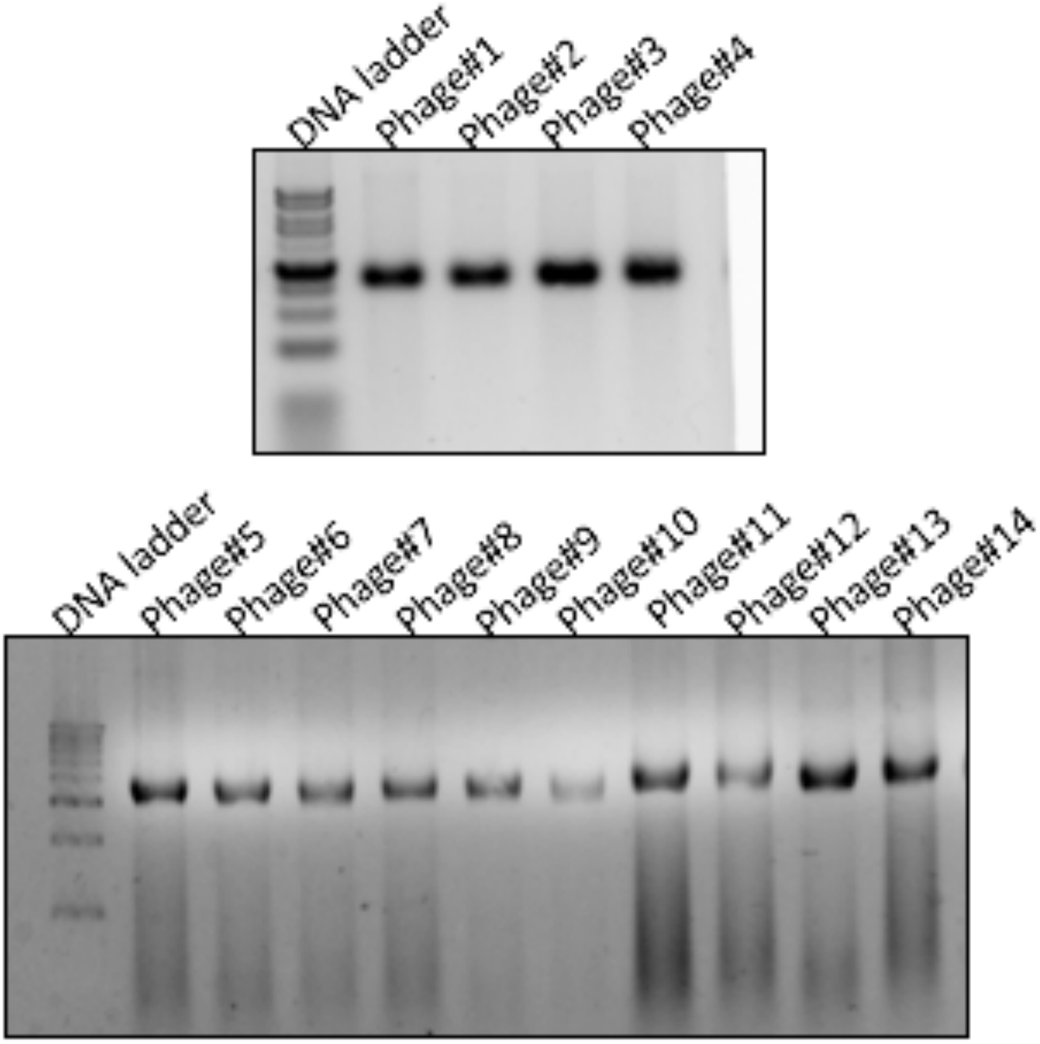

Figure S1. Purified phage DNA for ligand sequence analysis. Upper panel shows the purified DNA of 7-mer ligand expressing phages (phage#1 to phage#4) and interaction with Rv1523 proteins in second round of panning. Lower panel shows the purified DNA of 7-mer ligand expressing phages (phage#5 to phage#14) and interaction with Rv1523 proteins in third round of panning. The sequence of ligands were analysed and BLAST analyses carried out against the *M.tb* proteome. The interacting partners are enlisted in Table 1.

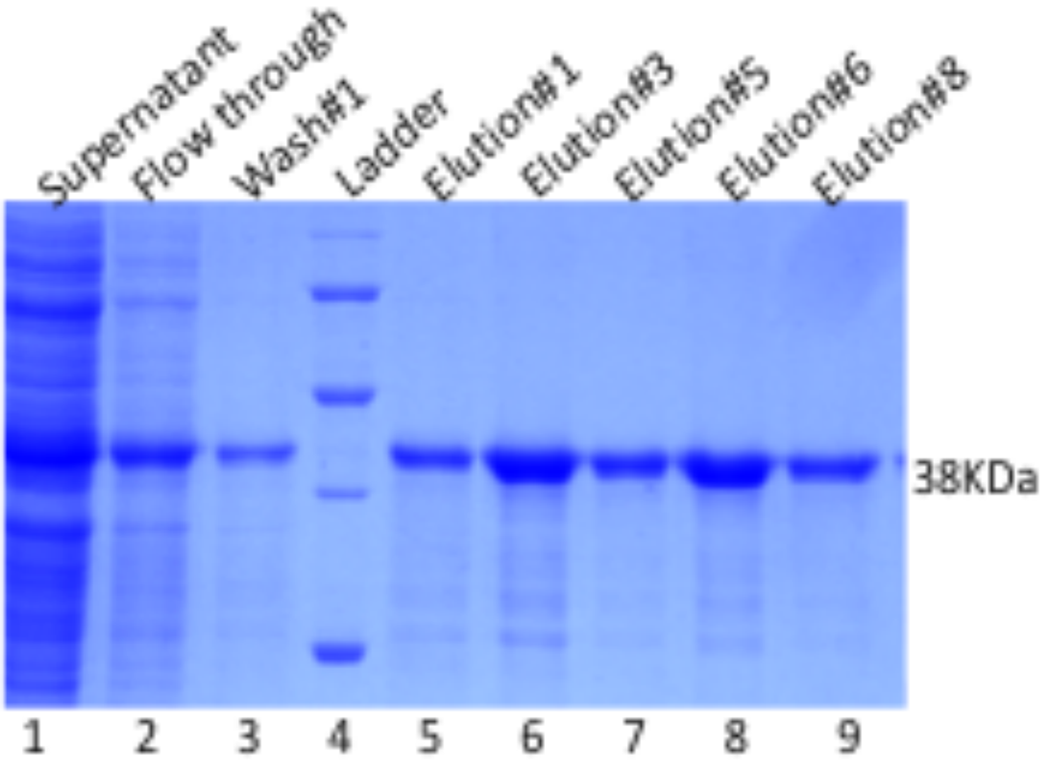

Figure S2. Expression and purification of Rv1523 protein. Purification of protein Rv1523, where Lane 1 – supernatant of culture; Lane 2- Flow through; Lane 3- wash fractions; Lane 4 - protein marker; Lanes 5-9 - the eluted fractions of the protein; (B) Purification of protein Rv2952, where Lane 1 – supernatant of culture; Lane 2- protein marker; Lane 3- Flow through; Lane 4 - wash fractions; Lanes 5-9 - the eluted fractions of the protein

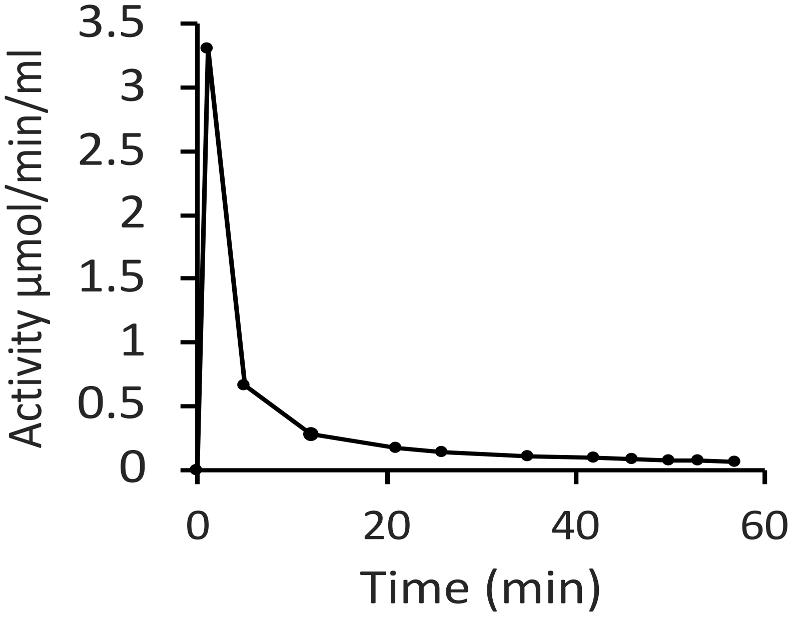

Figure S3. (A) Assay for Rv1523 enzyme activity with time was measured using 50ng Rv1523 protein, cell wall component FAMEs and MAMEs and SAM as methyl group donor in a colorimetric based assay.

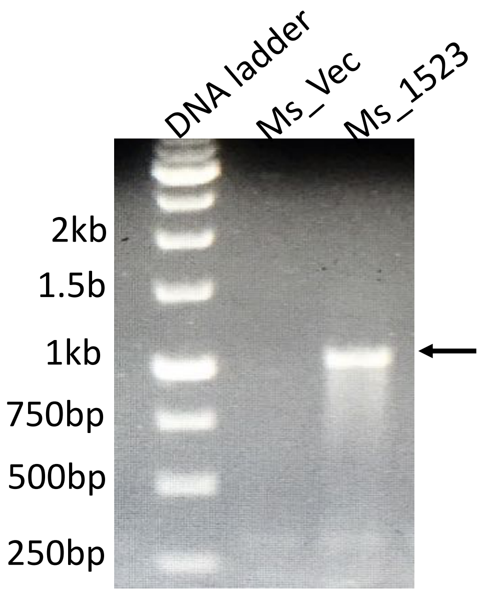

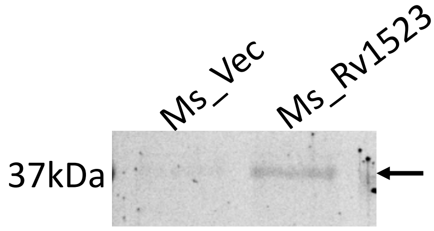

Figure S3. (B) Heterologous expression of *M. tuberculosis* Rv1523 in *M. smegmatis*, Ms_Vec and Ms_Rv1523 were grown into an OD_{600} of 1 and DNA isolated from them was subjected to PCR amplification to detect Rv1523 genein the electroporated *M. smegmatis*. Arrow indicates the presence of Rv1523 gene in Ms_Rv1523.

Figure S3. (C) The cell lysates prepared from Ms_Vec and Ms_Rv1523 were subjected to Western blot to determine the expression of Rv1523 protein in *M. smegmatis* by anti- Rv1523

antibodies raised in rabbit. No expression of Rv1523 protein was seen in Ms_Vec. Arrow shows Rv1523 protein expression in Ms_Rv1523.

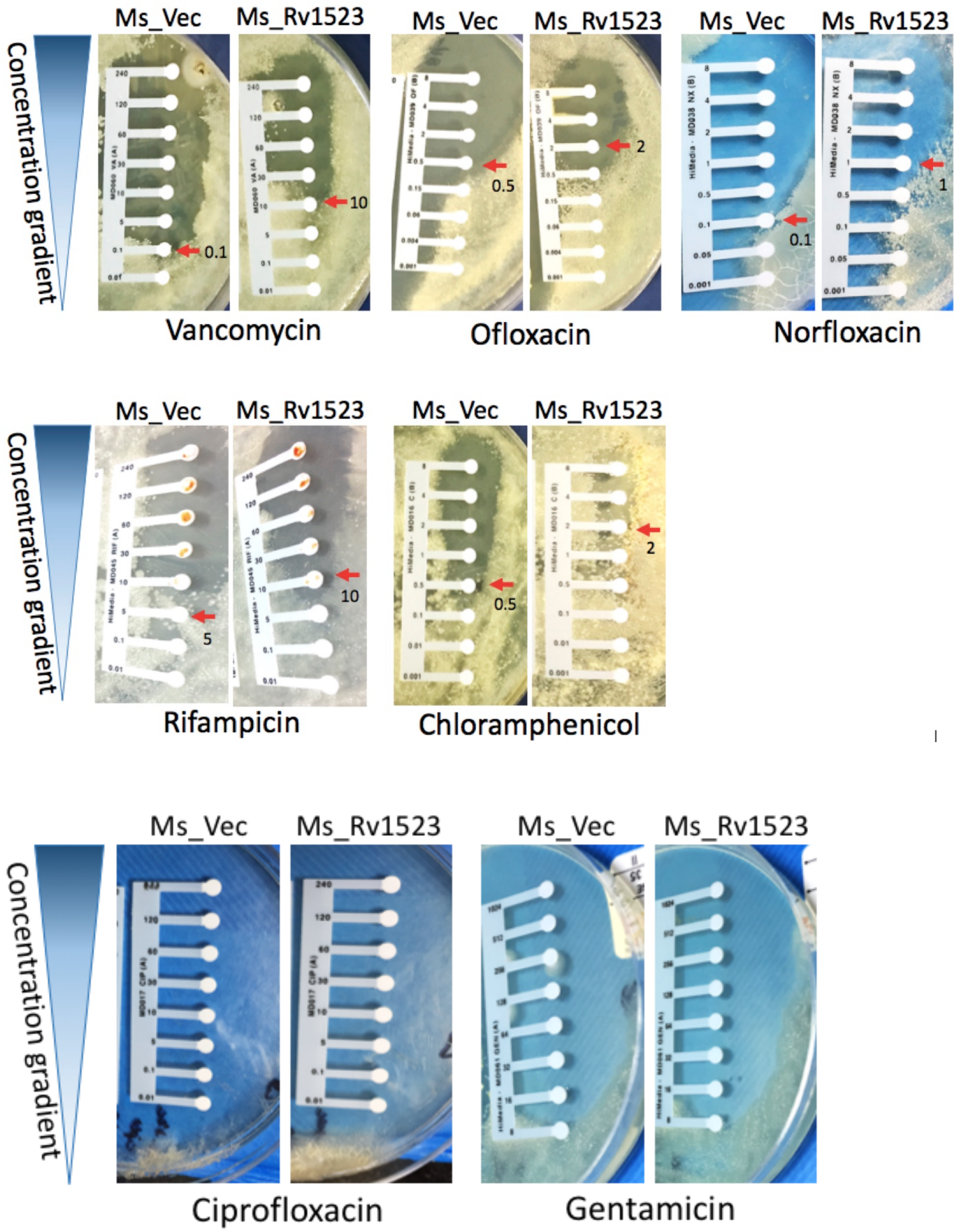

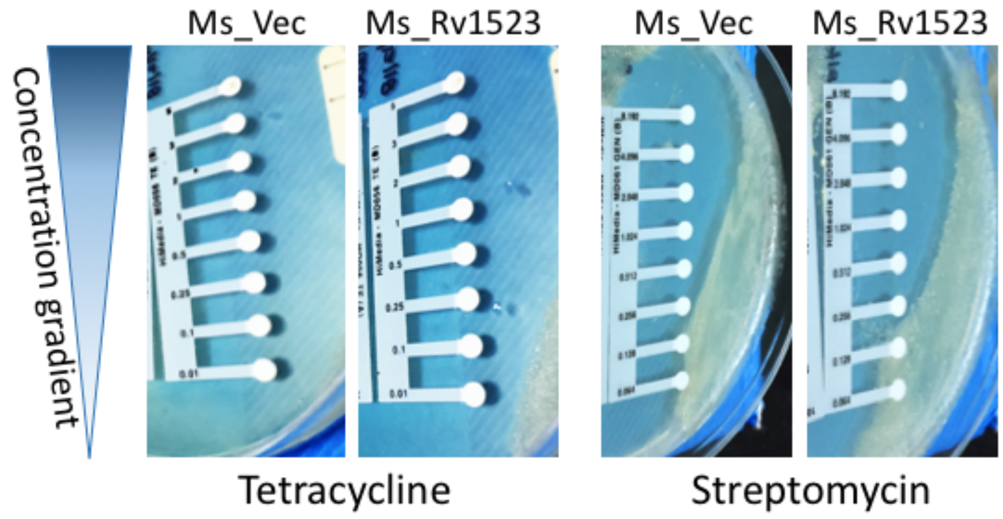

Figure S4. Recombinant Ms_Rv1523 and Ms_Vec were treated with nine anti-tuberculosis drugs and MIC was detected. Recombinant Ms_Vec and Ms_Rv1523 were grown till OD_{600} reached 0.06-0.08, the bacterial culture was diluted 2-fold and 50μl of each strain was plated into 7H10 agar medium and the HiComb^{TM} MIC Test strip was placed on the culture. HiComb^{TM} MIC Test strip provides a set of 16 different concentrations in gradient that can be easily used to deduce a functionally accurate Minimum Inhibitory Concentration (MIC) in microgram levels. MIC values of each antibiotic were assessed by analysing the bacterial growth after 3 days culture. Two upper panels show Ms_Rv1523 and Ms_Vec treated with HiComb^{TM} MIC Test strip of. Two lower panels show Ms_Rv1523 and Ms_Vec treated with HiComb^{TM} MIC Test strip of Tetracycline (left), and Streptomycin (right). Both Ms_1523 and Ms_Vec displayed comparable susceptibility to ciprofloxacin (Cip), gentamicin (Gen), tetracycline (Tet), and streptomycin (Str). Clear zone indicates that neither Ms_Rv1523 nor Ms_Vec were able to survive in the presence of Cip, Gen, Tet and Str. Albeit very less growth in every case can be seen at the edges of the culture plate.
