# Supplementary material for: The M. tuberculosis Rv1523 Methyltransferase Promotes Drug Resistance Through Methylation-Mediated Cell Wall Remodeling and Modulates Macrophages Immune Responses
Source: Front Cell Infect Microbiol. 2021 Mar 12;11:622487. doi: 10.3389/fcimb.2021.622487 (PMC7994892; doi:10.3389/fcimb.2021.622487)
Supplement: Supplementary file 2 [file DataSheet_2.pdf]

Supplementary Table. 1. Strains used in this study and their description.

Strain Used	Description
**WT**	Wild type *M. smegmatis* mc^2 155 strain.
**Ms_Vec**	*M. smegmatis* transformed with vector alone pVV16.
**Ms_Rv1523**	*M. smegmatis* with transformed with vector pVV16_Rv1523.
**Rv1523 DH5-α *E. coli***	Strain used in vector proliferation.
**Rv1523 BL21 *E. coli***	Strain used for Rv1523 protein purification.

Supplementary Table. 2. Plasmids used in this study and their description.

Plasmids	Description
**pVV16**	A replicative plasmid used for gene expression in *M. smegmatis* and conferring kanamycin(kan) and hygromycin (hyg) resistance.
**pET28**	A replicative plasmid with His-tag used for gene expression and purification in E. coli and conferring kanamycin(kan) resistance.

Supplementary Table. 3. Peptide Ligand for Rv1523

**S.N.**	**Peptide ligand**
**2nd Round**	
Phage#1	ADARYKS
Phage#2	HWNTVVS
Phage#3	DRGHHIL
Phage#4	MPRLPPA
**3rd Round**	
Phage#5	AWPYVTL
Phage#6	ISTTLFP
Phage#7	NTALSST
Phage#8	ADARYKS
Phage#9	LQGKGMT
Phage#10	SHLNVHS
Phage#11	ASSHIHH
Phage#12	NGATYPS
Phage#13	ADARYKS
